# Systematic review and meta-analysis of safety of laparoscopic *versus* open appendicectomy for suspected appendicitis in pregnancy

**DOI:** 10.1002/bjs.8889

**Published:** 2012-11

**Authors:** C Wilasrusmee, B Sukrat, M McEvoy, J Attia, A Thakkinstian

**Affiliations:** 1 Department of Surgery, Section for Clinical Epidemiology and Biostatistics, Faculty of MedicineRamathibodi Hospital, Mahidol University, Bangkok, Thailand; 2 Section for Clinical Epidemiology and Biostatistics, Faculty of Medicine, Ramathibodi Hospital, Mahidol UniversityBangkok, Thailand; 3 Bureau of Reproductive Health, Department of Health, Ministry of Public HealthNonthaburi, Thailand; 4 Centre for Clinical Epidemiology and Biostatistics, Newcastle UniversityNewcastle, New South Wales, Australia

## Abstract

**Background:**

Laparoscopic appendicectomy has gained wide acceptance as an alternative to open appendicectomy during pregnancy. However, data regarding the safety and optimal surgical approach to appendicitis in pregnancy are still controversial.

**Methods:**

This was a systematic review and meta-analysis of studies comparing laparoscopic and open appendicectomy in pregnancy identified using PubMed and Scopus search engines from January 1990 to July 2011. Two reviewers independently extracted data on fetal loss, preterm delivery, wound infection, duration of operation, hospital stay, Apgar score and birth weight between laparoscopic and open appendicectomy groups.

**Results:**

Eleven studies with a total of 3415 women (599 in laparoscopic and 2816 in open group) were included in the analysis. Fetal loss was statistically significantly worse in those who underwent laparoscopy compared with open appendicectomy; the pooled relative risk (RR) was 1·91 (95 per cent confidence interval (c.i.) 1·31 to 2·77) without heterogeneity. The pooled RR for preterm labour was 1·44 (0·68 to 3·06), but this risk was not statistically significant. The mean difference in length of hospital stay was − 0·49 (−1·76 to − 0·78) days, but this was not clinically significant. No significant difference was found for wound infection, birth weight, duration of operation or Apgar score.

**Conclusion:**

The available low-grade evidence suggests that laparoscopic appendicectomy in pregnant women might be associated with a greater risk of fetal loss. Copyright © 2012 British Journal of Surgery Society Ltd. Published by John Wiley & Sons, Ltd.

## Introduction

Suspected appendicitis is the most common indication for surgery for non-obstetric conditions during pregnancy, and occurs in approximately one in 635 to one in 500 pregnancies per year^1^, ^2^. Appendicitis occurs more frequently in the second trimester than in the first or third trimester of pregnancy^2–6^. Abdominal surgery during pregnancy, particularly appendicectomy^7^, may increase the risk of poor pregnancy outcomes^8^. Fetal loss usually occurs in 3–15 per cent of women with complicated appendicectomy during the first trimester. However, the rate may be as high as 20–30 per cent^9–11^, with a premature delivery rate of 15–45 per cent^12^, and a significantly increased risk of spontaneous abortion, premature labour, and perinatal morbidity and mortality^13^. Miscarriage and infant mortality occur more frequently in women with perforated appendicitis^14^. However, the maternal mortality rate is very low^15^, ^16^ as a result of the use of advanced antibiotics, close perioperative monitoring, cooperation between specialties and improvements in perioperative management^17^, ^18^.

Although guidelines for laparoscopic procedures during pregnancy have been established^19^, concern remains over the safety of the procedure, with reports of an increased risk of intra-abdominal abscess, particularly in perforated appendicitis. Assessment for open appendicectomy is related to gestational age as the appendix progressively relocates during pregnancy, typically from McBurney's point upwards from the iliac crest to near the gallbladder. Open appendicectomy is an established and safe operation with acceptable morbidity and low mortality rates.

Previous studies^11^, ^13–15^, ^17^, ^18^ were underpowered to detect any benefit of laparoscopic appendicectomy over the traditional open approach, resulting in conflicting results regarding the efficacy of laparoscopic appendicectomy for appendicitis during pregnancy. Only one randomized clinical trial comparing laparoscopic with open appendicectomy in pregnant women has been performed, with quality of life as the primary outcome^20^. One previous systematic review, including 28 observational studies that documented 637 laparoscopic appendicectomies, suggested that the laparoscopic procedure was associated with a higher rate of fetal loss but a similar or lower rate of preterm delivery compared with open appendicectomy^21^. However, the magnitude of treatment effects was not quantified. A systematic review and meta-analysis was therefore carried out, with the primary aim of estimating and comparing pregnancy outcomes including rates of fetal loss, preterm delivery, Apgar score and low birth weight.

## Methods

### Study selection

Studies published between January 1990 and 11 July 2011 were identified from MEDLINE and Scopus databases using PubMed and Scopus search engines respectively. Search terms used were: pregnancy, pregnant women, laparoscopy, laparoscopic appendectomy, laparoscopic management, open appendectomy, conventional appendectomy, maternal outcome, premature labor pain, preterm labor, abortion, fetal loss, gestational age, fetal outcome, birth weight, Apgar score, surgical outcome, hospital stay, length of stay, hospitalization length, operative time, operation time, duration of operation, infection, wound infection, surgical infection and negative appendectomy. Search strategies are described in *Table S1* (supporting information).

### Inclusion criteria

Studies were included in the review if they met the following criteria: studied patients were pregnant women with suspected appendicitis; the intervention and comparator were laparoscopic and open appendicectomy respectively; at least one pregnancy (for example preterm delivery, fetal loss, birth weight, Apgar score) or surgical (duration of operation, length of hospital stay, wound infection, negative appendicectomy) outcome was reported; and the study was published in English. Studies were excluded if a hybrid procedure or single-trocar technique was used rather than the standard laparoscopic appendicectomy.

The reference lists of all relevant studies were also reviewed. If studies were duplicates, the one with the most complete data was included. For studies that reported insufficient data, the corresponding authors were contacted and invited to provide more information. Two attempts were made to contact authors and if no response was obtained the study was excluded from the review.

### Outcomes

The primary outcomes of interest were the pregnancy outcomes fetal loss and preterm delivery. Secondary outcomes were: birth weight, Apgar score and surgical outcomes, including duration of operation, length of hospital stay, wound infection and negative appendicectomy (appendicitis not proven pathologically).

### Data extraction

Two investigators independently extracted the data from each study using a standard data extraction form. Information extracted included general data (author, year of publication, journal), study characteristics (study design, setting), patient characteristics (age, gestational age at surgery, gravida, body temperature, white blood cell count, number of subjects per group), and outcome as described above. Any disagreement was discussed and resolved by consensus with a third reviewer.

### Assessment of risk of bias

The quality of studies was assessed independently by three reviewers on the basis of representativeness of studied subjects, information bias (ascertainment of outcome and interventions) and confounding bias (*Table S2*, supporting information). Each item was graded as having a low risk of bias, a high risk of bias, or an unclear risk if there was insufficient information to judge^22^. Any disagreement between the reviewers was discussed and resolved by consensus.

### Statistical analysis

All analyses were performed using Stata version 11.1 (StataCorp LP, College Station, Texas, USA)^23^. For dichotomous outcomes (preterm delivery, fetal loss and wound infection), the relative risk (RR) of the outcome between laparoscopic *versus* open appendicectomy and its 95 per cent confidence interval (c.i.) were estimated for each study. If one cell in the 2 × 2 table contained zero, a continuity correction was performed by adding 0·5 to each cell^24^. Heterogeneity of RRs across studies was assessed using Cochran's Q test and the degree of heterogeneity was estimated using the *I*^2^ statistic. If the heterogeneity was significant or *I*^2^ exceeded 25 per cent, a random-effects model using the DerSimonian and Laird method was applied for pooling ORs; otherwise the inverse variance method was used^25^, ^26^.

For continuous variables (duration of operation, length of hospital stay, Apgar score, birth weight) the unstandardized mean difference in outcomes between groups along with its 95 per cent c.i. was estimated and the values were pooled. Heterogeneity of the mean difference across studies was assessed as described above.

Meta-regression analysis was used to assess the source of heterogeneity by fitting age and gestational age at surgery in the meta-regression model. A funnel plot with or without contour enhancement was used to detect publication bias owing to small study effects. The asymmetry of the funnel plot was assessed by means of Egger's test. The trim-and-fill method was used to impute missing studies if there was evidence of asymmetry of the funnel. *P* < 0·050 was considered statistically significant, except for the heterogeneity test, for which *P* < 0·100 was used.

## Results

**Fig 1:**
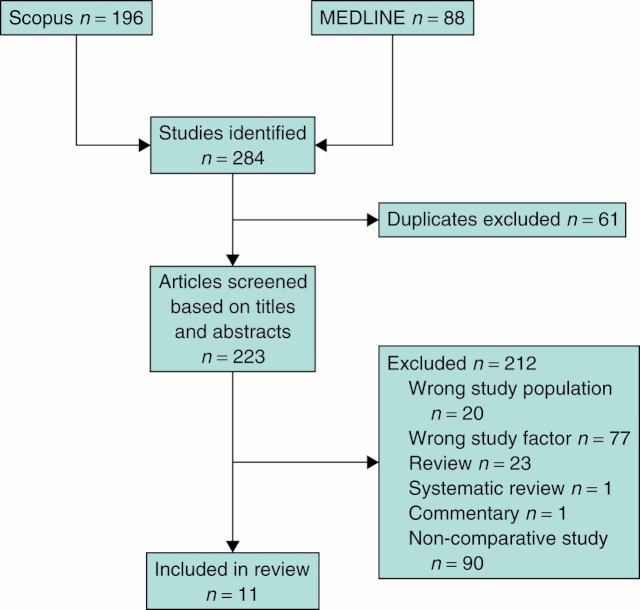
Identification of studies for inclusion in review

The initial literature search identified 88 and 196 studies from MEDLINE and Scopus databases respectively. Sixty-one studies were duplicates, leaving 223 for title or abstract review. After exclusion of 212 ineligible articles, 11 studies remained for analysis (*Fig. 1*). Agreement in data extraction between the two reviewers was 93·9 per cent (κ = 0·93, *P* < 0·001) and 92·2 per cent (κ = 0·0·92, *P* = 0 < 0·001) for dichotomous and continuous outcomes respectively.

The 11 included studies contained a total of 3415 patients (599 in laparoscopic and 2816 in open group) (Table 1)^12^, ^27–36^. Eight studies were comparative prospective cohort studies and three were comparative retrospective medical record reviews. Nine of the 11 studies were from the USA. The mean patient age ranged from 23·4 to 29·5 years. Gestational age at surgery was mostly in the second trimester, except in the study by Upadhyay and colleagues^30^. Four studies reported failure of laparoscopic appendicectomy, and the need to convert to open surgery in between one and three patients in each study^27^, ^29^, ^33^, ^36^. Two of these studies carried out intention-to-treat analysis, including the patient in the laparoscopic group^27^, ^29^. One study applied a per-protocol analysis^33^ and the other excluded patients whose operation was converted^36^.

**Table 1:** Baseline characteristics of included studies

						No. of women		
Reference	Year	No. of women	Age (years)*	Gestational age (weeks)*	Negative appendicectomy (%)	Laparoscopic	Open	Outcomes
Corneille *et al.*^27^	2010	49	25·6(6·4)	15·9(8·4)	NA	9	40	Fetal loss, hospital stay, preterm delivery
Sadot *et al.*^28^	2010	57	29·5(5·9)	19·7(7·2)	24	41	16	Apgar score, birth weight, fetal loss, hospital stay, duration of operation, preterm delivery, wound infection
Kirshtein *et al.*^29^	2009	42	28·4	13·9(6)	12	23	19	Apgar score, birth weight, fetal loss, hospital stay, duration of operation, wound infection
McGory *et al.*^12^	2007	3133	NA	NA	23·1	454	2679	Fetal loss
Upadhyay *et al.*^30^	2007	6	27·2(3·3)	32(2·6)	17	4	2	Fetal loss, preterm delivery
Carver *et al.*^31^	2005	28	23·4(5·8)	14(5·4)	NA	17	11	Apgar score, birth weight, fetal loss, hospital stay, preterm delivery, wound infection
Lyass *et al.*^32^	2001	22	28·5(15·2)	20(6·3)	NA	11	11	Fetal loss, hospital stay, duration of operation, preterm delivery
Affleck *et al.*^33^	1999	37	NA	NA	NA	19	18	Fetal loss, preterm delivery
Conron *et al.*^34^	1999	21	NA	NA	NA	12†	9‡	Apgar score, birth weight, fetal loss, hospital stay, duration of operation
Gurbuz and Peetz^35^	1997	9	24·5(1·5)	20·1(9)	22	5	4	Fetal loss, hospital stay, duration of operation, preterm delivery
Curet *et al.*^36^	1996	11	NA	NA	NA	4	7	Fetal loss

* Values are mean(s.d.).

† Includes laparoscopic cholecystectomy;

‡ includes open cholecystectomy. NA, not available.

The negative appendicectomy rate ranged from 12 to 24 per cent. Fetal loss was reported in all 11 studies^12^, ^27–36^ and preterm labour in seven^27^, ^28^, ^30–33^, ^35^. Four studies reported on Apgar scores^28^, ^29^, ^31^, ^34^, but only three had sufficient data to pool^28^, ^31^, ^34^, and four reported birth weight^28^, ^29^, ^31^, ^34^.

### Risk of bias

Assessment of risk of bias is reported in Table 2. The agreement between two reviewers was 95·5 per cent with a κ statistic of 0·94 (*P* < 0·001). Among 11 studies, the risk of selection bias from the use of non-representative cases was low in seven and unclear in four studies. The ascertainment of all outcomes was clearly described (except for wound infection) in six studies. Ascertainment of surgical technique was clear in seven studies. Unclear ascertainment in four studies was due to conversion from laparoscopic appendicectomy to an open technique. Confounding bias was likely to be present in ten studies.

**Table 2:** Quality assessment of included studies

Reference	Representativeness of cohorts	Ascertainment of outcome	Ascertainment of intervention	Confounding bias	Note
Corneille *et al.*^27^	Low risk	Low risk*	High risk	High risk	2 operations in LA group converted to OA
Sadot *et al.*^28^	Low risk	Low risk*	Low risk	High risk	
Kirshtein *et al.*^29^	Low risk	Low risk	High risk	High risk	1 operation in LA group converted to OA
McGory *et al.*^12^	Low risk	Low risk*	Low risk	Low risk	Applied logistic regression, adjusted for age and race
Upadhyay *et al.*^30^	Unclear	Low risk*	Low risk	High risk	
Carver *et al.*^31^	Low risk	Unclear	Low risk	High risk	
Lyass *et al.*^32^	Low risk	Unclear	Low risk	High risk	
Affleck *et al.*^33^	Low risk	Low risk*	High risk	High risk	2 operations in LA group converted to OA
Conron *et al.*^34^	Unclear	Unclear	Low risk	High risk	
Gurbuz and Peetz^35^	Unclear	Unclear	Low risk	High risk	
Curet *et al.*^36^	Unclear	Unclear	High risk	High risk	3 operations in LA group converted to OA

* Except wound infection. LA, laparosopic appendicectomy; OA, open appendicectomy.

### Fetal loss

**Fig 2:**
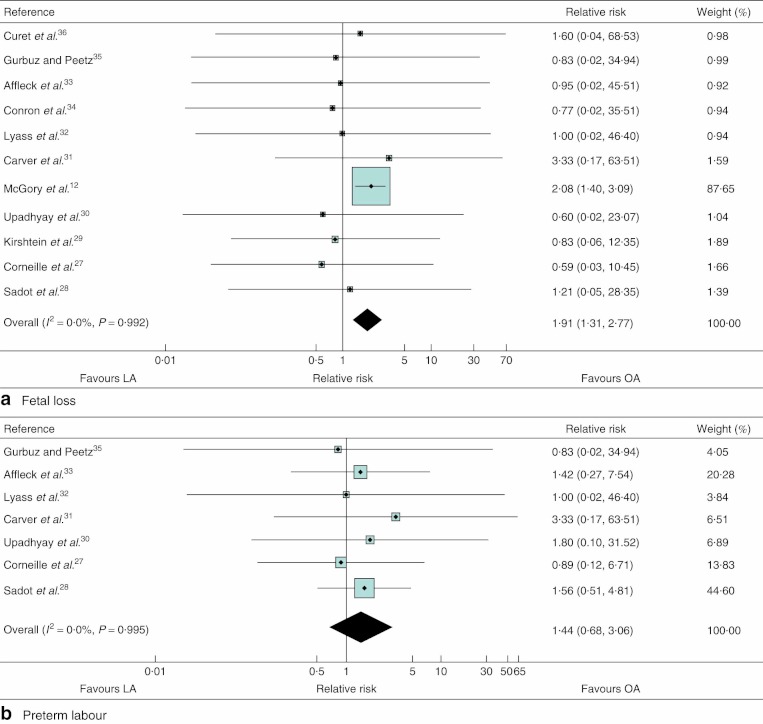
Meta-analysis of pregnancy outcomes a fetal loss and b preterm labour after laparoscopic (LA) *versus* open (OA) appendicectomy. Relative risks are shown with 95 per cent confidence intervals

All 11 studies (3415 women) reported fetal loss after appendicectomy^12^, ^27–36^, which allowed quantitative pooled analysis. The RRs were homogeneous (χ^2^ = 2·44, 10 d.f., *P* = 0·992; *I*^2^ = 0 per cent) with a pooled value (laparoscopic *versus* open appendicectomy) of 1·91 (95 per cent c.i. 1·31 to 2·77) (Table 3, Fig. 2a). This suggested that the odds of fetal loss was almost twice as high in the laparoscopy group as in the open appendicectomy group.

**Table 3:** Comparisons of fetal loss and preterm labour between laparoscopic and open appendicectomy in pregnancy

	Laparoscopic		Open		
Reference	Yes	No	Yes	No	Relative risk
Fetal loss					
Corneille *et al.*^27^	0	9	3	37	0·59 (0·03, 10·45)
Sadot *et al.*^28^	1	40	0	16	1·21 (0·05, 28·35)
Kirshtein *et al.*^29^	1	22	1	18	0·83 (0·06, 12·35)
McGory *et al.*^12^	31	423	88	2591	2·08 (1·40, 3·09)
Upadhyay *et al.*^30^	0	4	0	2	0·60 (0·02, 23·07)
Carver *et al.*^31^	2	15	0	11	3·33 (0·17, 63·51)
Lyass *et al.*^32^	0	11	0	11	1·00 (0·02, 46·40)
Affleck *et al.*^33^	0	19	0	18	0·95 (0·02, 45·51)
Conron *et al.*^34^	0	12	0	9	0·77 (0·02, 35·51)
Gurbuz and Peetz^35^	0	5	0	4	0·83 (0·02, 34·94)
Curet *et al.*^36^	0	4	0	7	1·60 (0·04, 68·53)
Pooled relative risk					1·91 (1·31, 2·77)
Preterm labour					
Corneille *et al.*^27^	1	8	5	35	0·89 (0·12, 6·71)
Sadot *et al.*^28^	12	29	3	13	1·56 (0·51, 4·81)
Upadhyay *et al.*^30^	1	3	0	2	1·80 (0·10, 31·52)
Carver *et al.*^31^	2	15	0	11	3·33 (0·17, 63·51)
Lyass *et al.*^32^	0	11	0	11	1·00 (0·02, 46·40)
Affleck *et al.*^33^	3	16	2	16	1·42 (0·27, 7·54)
Gurbuz and Peetz^35^	0	5	0	4	0·83 (0·02, 34·94)
Pooled relative risk					1·44 (0·68, 3·06)

Values in parentheses are 95 per cent confidence intervals.

**Fig 3:**
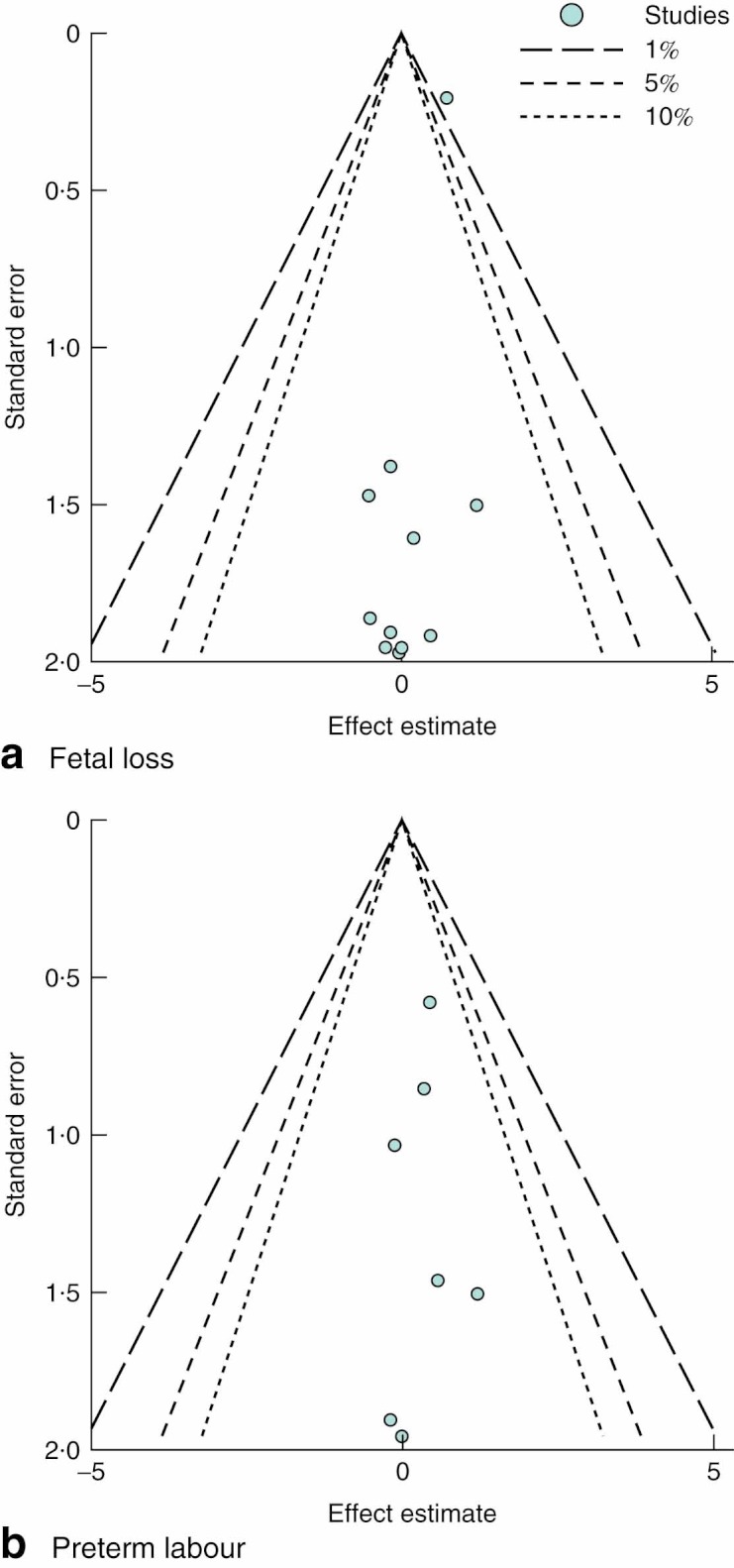
Contour-enhanced funnel plots for studies comparing a fetal loss and b preterm labour after laparoscopic *versus* open appendicectomy

Egger's test suggested asymmetry of the funnel (coefficient − 0·47, s.e. 0·13, *P* = 0·005). A contour-enhanced funnel plot was therefore created (Fig. 3a); this showed that all studies were in the non-significant area except that by McGory and colleagues^12^ in which laparoscopic appendicectomy had a significantly higher risk. Despite this asymmetry, application of a non-parametric trim-and-fill method could not identify any missing study.

### Preterm delivery

Among seven studies (208 women) that reported preterm labour^27^, ^28^, ^30–33^, ^35^, the RRs were homogeneous across studies (χ^2^ = 0·69, 6 d.f., *P* = 1·000; *I*^2^ = 0 per cent) (Table 3). The pooled RR was 1·44 (0·68 to 3·06) (Fig. 2b), indicating that the odds of preterm labour was 44 per cent higher in the laparoscopy than the open appendicectomy group; however, this was not statistically significant. Egger's test did not suggest publication bias (coefficient − 0·89, s.e. 0·34, *P* = 0·802) and this was supported by a symmetrical contour-enhanced funnel plot (Fig. 3b).

### Other pregnancy outcomes

Among four studies that reported birth weight (*n* = 148)^28^, ^29^, ^31^, ^34^, there was no heterogeneity (χ^2^ = 0·66, 3 d.f., *P* = 0·882; *I*^2^ = 0 per cent). The unstandardized pooled mean difference was 0·06 (95 per cent c.i. − 0·05 to 0·16) kg, suggesting that birth weights were similar in the two groups (Table 4). Apgar scores in the laparoscopic and open appendicectomy groups were compared in three studies (*n* = 106)^28^, ^31^, ^34^. The data were heterogeneous (χ^2^ = 9·70, 2 d.f., *P* = 0·008; *I*^2^ = 78·6 per cent), with an unstandardized mean difference of 0·05 (−0·18 to 0·27), indicating no significant different in Apgar scores between groups (Table 4).

**Table 4:** Comparison of secondary outcomes between laparoscopic and open appendicectomy

		No. of women			
	No. of included studies	Laparoscopic	Open	*I*^2^ (%)	Pooled effect*
Birth weight (kg)	4	93	55	0	0·06 (−0·05, 0·16)
Apgar score	3	70	36	78·6	0·05 (−0·18, 0·27)
Wound infection	3	81	46	0	0·91 (0·12, 7·18)
Duration of operation (min)	5	92	59	59·5	5·88 (−1·58, 13·33)
Hospital stay (days)	7	118	110	90·9	− 0·49 (−1·76, − 0·78)

Values in parentheses are 95 per cent confidence intervals.

* Pooled relative risk for wound infection and pooled mean difference for other outcomes.

### Surgical outcomes

Wound infection, duration of operation and hospital stay were also pooled across studies (Table 4). Pooling wound infection in three studies (*n* = 127)^28^, ^29^, ^31^ yielded a pooled RR of 0·91 (0·12 to 7·18), suggesting little difference in the risk of wound infection between interventions. The duration of operation was longer in the laparoscopy group, by a mean of 5·88 (−1·58 to 13·33) min, but the difference was not significant. The length of hospital stay was significantly shorter in the laparoscopy group by almost half a day (95 per cent c.i. − 1·76 to − 0·78 days).

## Discussion

The results of this systematic review and meta-analysis suggest that laparoscopic appendicectomy in pregnancy results in an almost twofold significantly higher risk of fetal loss compared with open appendicectomy. No significant differences were observed between groups in preterm delivery, birth weight, Apgar score, wound infection after surgery or duration of operation.

The higher risk of fetal loss after laparoscopic compared with open appendicectomy needs to be addressed in the era of laparoscopic surgery, and has been discussed in many reports of the relative safety of laparoscopy in pregnancy^10^, ^13^, ^37^. However, this finding was largely dominated by the study of McGory and colleagues^12^, which had largest sample size and greatest power in detection of an association. After exclusion of this study from the pooled analysis, there was no effect of laparoscopic appendicectomy on fetal loss.

The major consideration in laparoscopic appendicectomy in pregnancy is the effect of increased intra-abdominal pressure and fetal acidosis during carbon dioxide pneumoperitoneum. Increasing abdominal pressure from the pneumoperitoneum can lead to decreased venous return, especially in women with impaired cardiac output^38^, and result in maternal hypotension and hypoxia^39^. In addition, it has been reported that carbon dioxide is also absorbed across the peritoneum, which leads to fetal acidosis^40^. However, this is in contrast the findings of another study that reported no substantial adverse effect on the fetus when the maximum pneumoperitoneal pressure was as high as 10–12 mmHg and the duration less than 30 min^41^.

Although not statistically significant, the present results suggest that there may be an increased risk of preterm delivery in those undergoing laparoscopic appendicectomy compared with open appendicectomy. It is likely that this analysis did not have sufficient statistical power to detect a significant difference, given that a sample size of 749 would be required in each group to detect a RR of 1·44.

Although the mean operating time was 5·88 min longer in the laparoscopic group, this was not statistically significant. The length of hospital stay was approximately half a day shorter after laparoscopic compared with the open appendicectomy, but this result depends heavily on one outlier study and cannot be considered robust. This requires further investigation for health service use planning, but a shorter hospital stay after a laparoscopic appendicectomy might not be advantageous clinically because of the need to monitor the patient for the adverse events noted above.

This meta-analysis quantified the effects of laparoscopic and open appendicectomy on pregnancy and surgical outcomes. A previous review did not pool data and most included studies were non-comparative, with only one group^21^. The present review included the most relevant pregnancy and surgical outcomes.

One major limitation is that all studies included in the pooled analysis were observational, and summary data published within each article were included in the review. Many other factors (such as patient age, duration of pregnancy, weight gain, complicated appendicitis, surgeon's skill, clinical setting) may affect the outcomes following appendicectomy, and confounding bias cannot be ruled out as the studies were not randomized. To adjust for confounding bias, individual patient data would be required from each study. There were no available data on complicated appendicitis (perforated and gangrenous) and it was not possible to assess whether the effects of laparoscopic appendicectomy on fetal loss were confounded by complicated appendicitis. Agreement between the present results and a meta-analysis of randomized trials or a subsequent large-scale trial is needed to confirm the present findings^42^. Given that pregnant women are subject to human-subject protection in clinical studies, it will be difficult to conduct a randomized trial. However, the authors believe that the direction of bias is probably conservative: those with more co-morbidity and who are considered high risk are likely to undergo open appendicectomy, making the laparoscopic approach look spuriously superior. The increased risk of fetal loss seen here is therefore likely to be an underestimate. It was not possible to identify a statistically significant difference for preterm delivery and infection owing to the limited number of studies available for pooling. Finally, as the severity of appendicitis was not reported consistently in the pooled studies, a subgroup analysis to identify specific subgroups of women who might benefit from, or be harmed by, laparoscopic appendicectomy was not possible.

## Supporting information

**Table S1** Search strategy for PubMed and Scopus (Word document) **Table S2** Assessment of risk of bias (Word document) Please note: John Wiley & Sons Ltd is not responsible for the functionality of any supporting materials supplied by the authors. Any queries (other than missing material) should be directed to the corresponding author for the article.

## References

[1] Kort B, Katz VL, Watson WJ (1993). The effect of nonobstetric operation during pregnancy. Surg Gynecol Obstet.

[2] Guttman R, Goldman RD, Koren G (2004). Appendicitis during pregnancy. Can Fam Physician.

[3] Gilo NB, Amini D, Landy HJ (2009). Appendicitis and cholecystitis in pregnancy. Clin Obstet Gynecol.

[4] Yilmaz HG, Akgun Y, Bac B, Celik Y (2007). Acute appendicitis in pregnancy—risk factors associated with principal outcomes: a case control study. Int J Surg.

[5] Ueberrueck T, Koch A, Meyer L, Hinkel M, Gastinger I (2004). Ninety-four appendectomies for suspected acute appendicitis during pregnancy. World J Surg.

[6] Rollins MD, Chan KJ, Price RR (2004). Laparoscopy for appendicitis and cholelithiasis during pregnancy: a new standard of care. Surg Endosc.

[7] Cohen HL, Moore WH (2004). History of emergency ultrasound. J Ultrasound Med.

[8] Al-Qudah MS, Amr M, Sroujieh A, Issa A (1999). Appendectomy in pregnancy: the experience of a university hospital. J Obstet Gynaecol.

[9] Andersen B, Nielsen TF (1999). Appendicitis in pregnancy: diagnosis, management and complications. Acta Obstet Gynecol Scand.

[10] Park SH, Park MI, Choi JS, Lee JH, Kim HO, Kim H (2010). Laparoscopic appendectomy performed during pregnancy by gynecological laparoscopists. Eur J Obstet Gynecol Reprod Biol.

[11] Palanivelu C, Rangarajan M, Senthilkumaran S, Parthasarathi R (2007). Safety and efficacy of laparoscopic surgery in pregnancy: experience of a single institution. J Laparoendosc Adv Surg Tech A.

[12] McGory ML, Zingmond DS, Tillou A, Hiatt JR, Ko CY, Cryer HM (2007). Negative appendectomy in pregnant women is associated with a substantial risk of fetal loss. J Am Coll Surg.

[13] Lemaire BMD, Van Erp WFM (1997). Laparoscopic surgery during pregnancy. Surg Endosc.

[14] Amos JD, Schorr SJ, Norman PF, Poole GV, Thomae KR, Mancino AT (1996). Laparoscopic surgery during pregnancy. Am J Surg.

[15] Halkic N, Tempia-Caliera AA, Ksontini R, Suter M, Delaloye JF, Vuilleumier H (2006). Laparoscopic management of appendicitis and symptomatic cholelithiasis during pregnancy. Langenbecks Arch Surg.

[16] Pastore PA, Loomis DM, Sauret J (2006). Appendicitis in pregnancy. J Am Board Fam Med.

[17] Wu JM, Chen KH, Lin HF, Tseng LM, Tseng SH, Huang SH (2005). Laparoscopic appendectomy in pregnancy. J Laparoendosc Adv Surg Tech A.

[18] Rizzo AG (2003). Laparoscopic surgery in pregnancy: long-term follow-up. J Laparoendosc Adv Surg Tech A.

[19] (1998). Guidelines for laparoscopic surgery during pregnancy. Surg Endosc.

[20] Kaplan M, Salman B, Yilmaz TU, Oguz M (2009). A quality of life comparison of laparoscopic and open approaches in acute appendicitis: a randomised prospective study. Acta Chir Belg.

[21] Walsh CA, Tang T, Walsh SR (2008). Laparoscopic *versus* open appendicectomy in pregnancy: a systematic review. Int J Surg.

[22] Higgins JPT, Green S (2008). Cochrane Handbook for Systematic Reviews of Interventions.

[23] Lu G, Ades AE (2004). Combination of direct and indirect evidence in mixed treatment comparisons. Stat Med.

[24] Fleiss JL (1993). The statistical basis of meta-analysis. Stat Methods Med Res.

[25] Higgins JP, Thompson SG, Deeks JJ, Altman DG (2003). Measuring inconsistency in meta-analyses. BMJ.

[26] Petitti DB (2001). Approaches to heterogeneity in meta-analysis. Stat Med.

[27] Corneille MG, Gallup TM, Bening T, Wolf SE, Brougher C, Myers JG (2010). The use of laparoscopic surgery in pregnancy: evaluation of safety and efficacy. Am J Surg.

[28] Sadot E, Telem DA, Arora M, Butala P, Nguyen SQ, Divino CM (2010). Laparoscopy: a safe approach to appendicitis during pregnancy. Surg Endosc.

[29] Kirshtein B, Perry ZH, Avinoach E, Mizrahi S, Lantsberg L (2009). Safety of laparoscopic appendectomy during pregnancy. World J Surg.

[30] Upadhyay A, Stanten S, Kazantsev G, Horoupian R, Stanten A (2007). Laparoscopic management of a nonobstetric emergency in the third trimester of pregnancy. Surg Endosc.

[31] Carver TW, Antevil J, Egan JC, Brown CVR (2005). Appendectomy during early pregnancy: what is the preferred surgical approach?. Am Surg.

[32] Lyass S, Pikarsky A, Eisenberg VH, Elchalal U, Schenker JG, Reissman P (2001). Is laparoscopic appendectomy safe in pregnant women?. Surg Endosc.

[33] Affleck DG, Handrahan DL, Egger MJ, Price RR (1999). The laparoscopic management of appendicitis and cholelithiasis during pregnancy. Am J Surg.

[34] Conron RW, Abbruzzi K, Cochrane SO, Sarno AJ, Cochrane PJ (1999). Laparoscopic procedures in pregnancy. Am Surg.

[35] Gurbuz AT, Peetz ME (1997). The acute abdomen in the pregnant patient. Is there a role for laparoscopy?. Surg Endosc.

[36] Curet MJ, Allen D, Josloff RK, Pitcher DE, Curet LB, Miscall BG (1996). Laparoscopy during pregnancy. Arch Surg.

[37] Lemieux P, Rheaume P, Levesque I, Bujold E, Brochu G (2009). Laparoscopic appendectomy in pregnant patients: a review of 45 cases. Surg Endosc.

[38] Westerband A, Van de Water JM, Amzallag M, Lebowitz PW, Nwasokwa ON, Chardavoyne R (1992). Cardiovascular changes during laparoscopic cholecystectomy. Surg Gynecol Obstet.

[39] Kammerer WS (1979). Nonobstetric surgery during pregnancy. Med Clin North Am.

[40] Soper NJ, Hunter JG, Petrie RH (1992). Laparoscopic cholecystectomy during pregnancy. Surg Endosc.

[41] Curet MJ, Vogt DA, Schob O, Qualls C, Izquierdo LA, Zucker KA (1996). Effects of CO_2_ pneumoperitoneum in pregnant ewes. J Surg Res.

[42] Ioannidis JP, Cappelleri JC, Lau J (1998). Issues in comparisons between meta-analyses and large trials. JAMA.

