# Supplementary material for: Systematic review and meta-analysis of safety of laparoscopic versus open appendicectomy for suspected appendicitis in pregnancy
Source: Br J Surg. 2012 Nov;99(11):1470–8. doi: 10.1002/bjs.8889 (PMC3494303; doi:10.1002/bjs.8889)
Supplement: Supplementary file 1 [file bjs0099-1470-SD1.doc]

**BJS8889**

**Systematic review and meta-analysis of safety of laparoscopic *versus* open appendicectomy for suspected appendicitis in pregnancy**

C. Wilasrusmee, B. Sukrat, M. McEvoy, J. Attia and A. Thakkinstian

**Table S1** Search strategy for PubMed and Scopus

1 pregnancy

2 pregnant women

3 laparoscopy

4 laparoscopic appendectomy

5 laparoscopic management

6 open appendectomy

7 conventional appendectomy

8 maternal outcome

9 premature labor pain

10 preterm labor

11 abortion

12 fetal loss

13 gestational age

14 fetal outcome

15 birth weight

16 APGAR score

17 surgical outcome

18 hospital stay

19 length of stay

20 hospitalization length

21 operative time

22 operation time

23 duration of operation

24 infection

25 wound infection

26 surgical infection

27 negative appendectomy

28 (1*or*2)

29 (3*or*4*or*5)

30 (6*or*7)

31 (8*or*7*or*10……………*or*27)

32 (28*and*29*and*30*and*31)

**Table S2** Assessment of risk of bias

| Domain | Item | Risk of bias |
| --- | --- | --- |
| Selection bias | Representativeness of cohorts  Consecutive or randomly selected cases from  population with clearly described inclusion criteria  Spectrum of diseases: negative appendicectomy less  than 25%  Did not mention | Low  Low  High |
| Information bias | Ascertainment of outcome  Clearly described definition of outcomes  Did not describe outcomes | Low  High |
|  | Ascertainment of intervention  No conversion (no failure of LA and conversion to OA)  At least one conversion  Not described | Low  High  Unclear |
| Confounding bias | Confounding bias  Adjusted for confounding factors in analysis  Did not adjust for confounding factors | Low risk  High risk |

LA, laparosopic appendicectomy; OA, open appendicectomy.
